# Chrysin-loaded PCL nanocapsule spray as a topical strategy against cutaneous candidiasis

**DOI:** 10.3389/fphar.2026.1975087

**Published:** 2026-10-15

**Authors:** Milena Luiza da Silva, Eric Luiz Domingos, Ahmad Kassem El Zein, Roberto Pontarolo, Marcel Henrique Marcondes Sari, Jéssica Brandão Reolon, Luana Mota Ferreira

**Affiliations:** 1 Center for Studies in Biopharmacy (CEB), Department of Pharmacy, Postgraduate Program in Pharmaceutical Sciences, Federal University of Paraná - UFPR, Curitiba, Brazil; 2 Metabolic Diseases Research Group (GPDM), Department of Clinical Analisys, Postgraduate Program in Pharmaceutical Sciences, Federal University of Paraná - UFPR, Curitiba, Brazil; 3 Postgraduate Program in Pharmaceutical Sciences, State University of Midwest (UNICENTRO), Guarapuava, Brazil

**Keywords:** 5,7-dihydroxyflavone, *Candida albicans*, chrysin, nanocapsules, skin candidiasis

## Abstract

**Introduction:**

*Candida albicans*, the most prevalent fungal pathogen in humans, poses increasing therapeutic challenges due to rising resistance to conventional azole antifungals, driving interest in natural alternatives. Chrysin (5,7- dihydroxyflavone) is a flavonoid with promising biological activities that are limited by poor aqueous solubility and low bioavailability. This study aimed to develop and characterize chrysin-loaded poly (ε-caprolactone) (PCL) nanocapsules in a spray formulation for topical treatment of cutaneous candidiasis.

**Methods:**

Nanocapsules were produced by interfacial deposition of the preformed polymer and characterized for particle size, zeta potential, encapsulation efficiency, and morphology. Their performance was further evaluated through in vitro release studies, antifungal activity against C. albicans, antioxidant activity, and spray performance.

**Results and Discussion:**

The formulation exhibited nanometric size (207.6 ± 4.63 nm), low polydispersity (0.174 ± 0.04), high encapsulation efficiency (87.28% ± 0.77%), and sustained drug release over 120 h, with antifungal activity substantially superior to free chrysin, requiring 5- to 8-fold lower concentrations to achieve equivalent inhibitory and fungicidal effects, and enhanced antioxidant efficacy at lower concentrations. The spray device delivered consistent and reproducible doses (118.5% of theoretical content, RSD 5.78%). These findings demonstrate that chrysin-loaded PCL nanocapsules successfully overcome solubility limitations, enhance antifungal and antioxidant activities, and provide sustained release, representing a promising nanotechnology-based alternative for cutaneous candidiasis treatment with convenient topical application.

## Graphical Abstract

**Figure:**
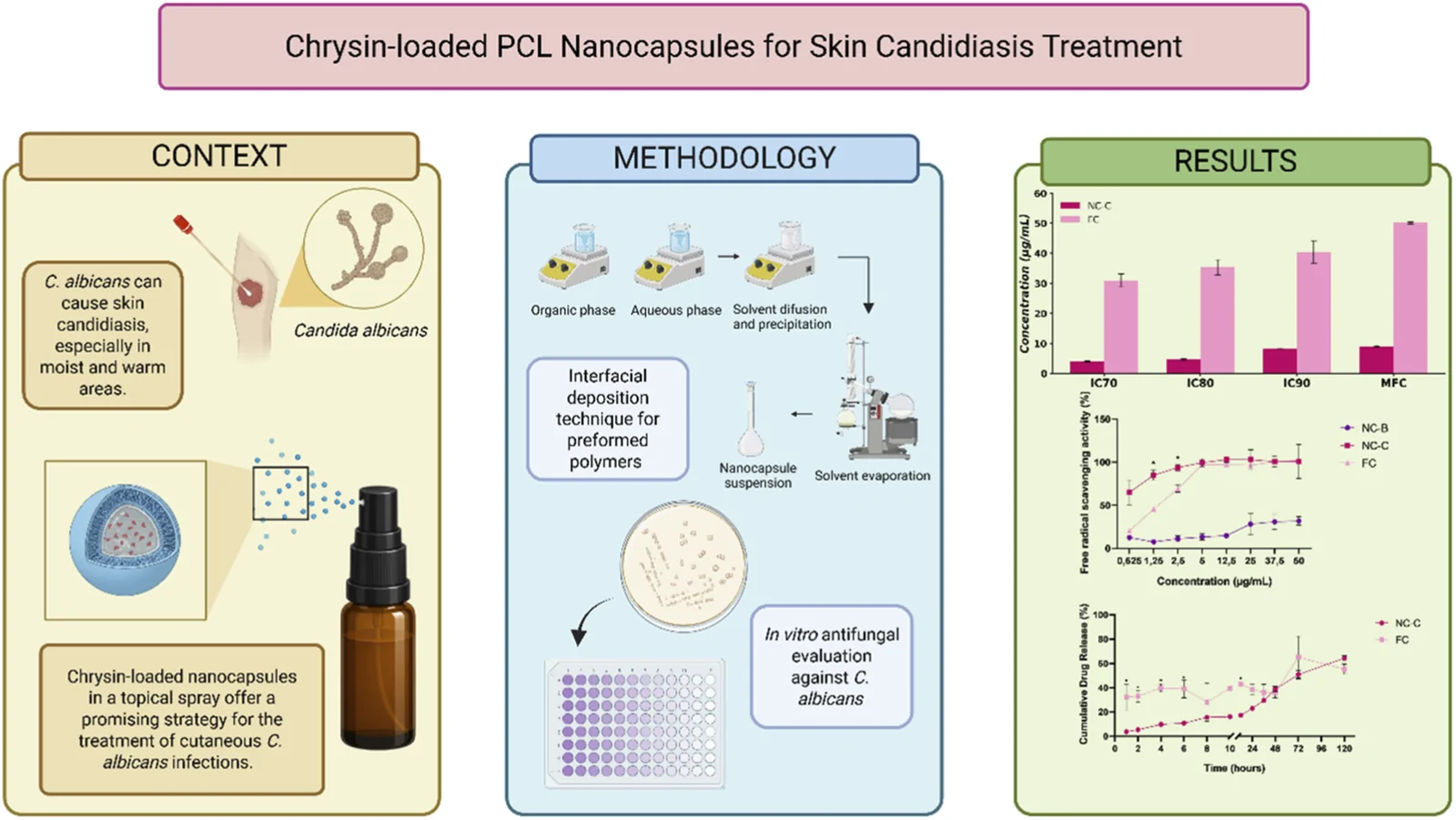

## 1 Introduction

*Candida albicans* is a ubiquitous polymorphic fungus that commonly exists as a commensal organism within the human microbiota, colonizing sites such as the oral cavity, skin, gastrointestinal tract, and vagina in the majority of healthy individuals (Shahabudin et al., 2024; Kashem and Kaplan, 2016). Nevertheless, it is recognized as an opportunistic pathogen, since disruptions to the equilibrium between the fungus and the host’s immune system can lead to conditions ranging from superficial infections to life-threatening invasive disease. It stands as the most prevalent fungal pathogen in humans, accounting for 70–90% of all fungal infections (Sharma et al., 2024; Talapko et al., 2021; Tong and Tang, 2017).

A key feature of *C. albicans* is its morphological plasticity, which enables it to transition between unicellular yeast forms, pseudohyphae, and true hyphae (Richardson and Moyes, 2015). Its virulence mechanism involves multiple factors, including: adhesins (surface proteins that promote fungal attachment to host cells and tissues); secretion of hydrolytic enzymes (aspartic proteases, phospholipases, and lipases) that degrade cell membranes and facilitate tissue invasion; production of the toxin candidalysin by hyphae, which causes membrane disruption and triggers an inflammatory response; and biofilm formation on both biotic and abiotic surfaces, which provides high resistance to antifungal agents and shields the fungus from immune defenses (Shahabudin et al., 2024; Talapko et al., 2021; Tong and Tang, 2017).

Cutaneous infections typically manifest in areas commonly exposed to moisture, heat, and friction. Common clinical presentations include intertrigo (infection of skin folds, such as the axillary or submammary areas), oral thrush (whitish-yellow lesions in the mouth and throat), diaper candidiasis (rashes in the genital and inner thigh regions of infants or incontinent adults), genital infections (vulvovaginal candidiasis in females and balanitis in males), and nail infections (onychomycosis and paronychia) (Shahabudin et al., 2024; Taudo et al., 2019; Espinosa-Hernández et al., 2020).

Standard treatment for *C. albicans* infections involves azole antifungals, such as clotrimazole or miconazole, administered as topical creams or as a single oral dose for uncomplicated cases. For more severe presentations or recurrent episodes, therapy initially consists of a topical or oral regimen lasting up to 14 days, followed by maintenance therapy with a weekly 150 mg dose of fluconazole for 6 months (Shahabudin et al., 2024).

Resistance to conventional antifungals is a growing concern in clinical practice, stemming from the widespread use and overprescription of agents like fluconazole. The mechanisms underlying resistance to azole antifungals occur primarily through overexpression of efflux pumps, which expel the drug from the fungal cell, or via mutations in the ERG11 gene, which lower the drug’s binding affinity for ergosterol, the therapeutic target of this drug class (Sharma et al., 2024). Because azoles are fungistatic rather than fungicidal, they also exert strong selective pressure on surviving fungal populations, making the resistance highly prevalent (Kessler et al., 2022). For instance, among diabetic patients with chronic skin ulcers, 66.6% of isolated *C. albicans* strains were resistant to fluconazole (Raiesi et al., 2017).

In the face of rising resistance to synthetic antifungals, natural products have emerged as a widely researched alternative, both as standalone therapies and as adjuvants to enhance the efficacy of existing drug classes. In this context, chrysin stands out as a compound of interest, given its broad spectrum of therapeutic activities and its demonstrated potential against microorganisms, including *C. albicans* (Candiracci et al., 2011; Parveen et al., 2023). Chrysin is a natural flavonoid widely found in honey, propolis, and medicinal plants such as *Passiflora* species and *Radix scutellariae* (Deldar et al., 2018; Nabavi et al., 2015). Structurally classified as 5, 7-dihydroxyflavone, it features the typical C2–C3 double bond of the flavone class and hydroxyl groups at positions 5 and 7, but lacks oxygenation in the B ring, a distinctive feature among flavonoids (Naz et al., 2019; Souza et al., 2015).

The pharmacological interest in chrysin stems from its broad bioactivity. It exhibits potent antioxidant effects through both direct free-radical scavenging and upregulation of endogenous enzymes such as SOD, CAT, and GSH-Px (Souza et al., 2015; Pushpavalli et al., 2010; da Silva et al., 2026). This oxidative stress modulation provides the basis for its neuroprotective properties, including benefits against Parkinson’s disease, depression, and cognitive decline (Goes et al., 2018; Del et al., 2021; Giacomeli et al., 2020). Additionally, chrysin exerts significant anti-inflammatory activity by reducing proinflammatory cytokines (IL-6, IL-1β, TNF-α) and suppressing COX-2 and iNOS expression (Deldar et al., 2018; Del Fabbro et al., 2025).

Despite this promising therapeutic potential, chrysin’s clinical application is critically hampered by its pronounced hydrophobicity. This leads to poor aqueous solubility, extensive intestinal and hepatic metabolism, low cellular uptake, and consequently, negligible oral bioavailability (Deldar et al., 2018; Nabavi et al., 2015). In other words, chrysin has the biological activity but lacks the means to reach its targets effectively.

Nanotechnology offers a compelling strategy to overcome these delivery barriers. Encapsulating chrysin within soft nanocarriers, such as polymeric or lipid nanoparticles, can convert its unfavorable physicochemical properties into favorable biopharmaceutical profiles, while also protecting the compound from premature chemical or enzymatic degradation (Estelrich et al., 2025; Malmsten, 2006). Furthermore, nanocarriers enable controlled release kinetics, prolonged retention at the target site, and can be functionalized with targeting ligands to achieve selective delivery to diseased tissues, thereby enhancing therapeutic efficacy and reducing systemic toxicity (Yang et al., 2020; Singh et al., 2025).

Structurally, polymeric nanocapsules are vesicular, core–shell delivery systems in which a liquid oily core, stabilized by a surfactant, is enclosed by a solid polymeric shell, an architecture that differs from matrix-type nanospheres, in which the drug is dispersed throughout a continuous polymer network (Lima et al., 2022). This configuration is particularly advantageous for lipophilic compounds such as chrysin, since the oily core provides a favorable microenvironment for drug solubilization, while the polymeric shell protects the payload and modulates its release (Mehandole et al., 2023). Poly (ε-caprolactone) (PCL) is among the most widely employed wall-forming polymers for this purpose, owing to its biodegradability, biocompatibility, and established regulatory approval for biomedical use (Ntrivala et al., 2025). Formulating this system also requires surfactants to stabilize the oil–water interface; lipophilic surfactants such as Span® 80 (sorbitan monooleate) reduce interfacial tension within the organic phase and support the formation of a stable oily core (Wu et al., 2025), while hydrophilic surfactants such as Tween® 80 (polysorbate 80) stabilize the resulting nanocapsule dispersion in the aqueous medium. Capryol® 90 (propylene glycol monocaprylate), in turn, is a medium-chain glyceride commonly used as the oily/solubilizing component of lipid-based nanocarriers, owing to its capacity to enhance the solubility and encapsulation of poorly water-soluble drugs (Sobhani et al., 2018).

From a pharmaceutical technology standpoint, incorporating nanocarrier suspensions into a spray delivery system offers a practical strategy for cutaneous administration. Spray devices atomize the liquid formulation into fine droplets, enabling non-contact application and a more uniform distribution of the drug over extensive, irregular, or friction-prone skin areas than manually spread creams or ointments, while also reducing the risk of secondary contamination and improving patient comfort and compliance (Kumar et al., 2026). This delivery format is increasingly explored for topical antifungal and dermatological formulations, as it combines the advantages of nanotechnology-based carriers with a convenient, patient-friendly mode of application.

Therefore, this study aimed to develop and characterize a chrysin-loaded PCL nanocapsule suspension, evaluate its antifungal activity against *C. albicans*, and propose a spray formulation for the treatment of cutaneous candidiasis.

## 2 Materials and methods

### 2.1 Materials

Chrysin, PCL, Span® 80, and Tween® 80 were purchased from Sigma-Aldrich (São Paulo, Brazil). Capryol® 90 was kindly donated by Gattefossé (Saint-Priest, France). All other solvents and reagents were analytical grade and used as received.

### 2.2 Analytical method

To determine the chrysin content, high-performance liquid chromatography (HPLC) analysis was performed using a method adapted from Liang et al. (Liang et al., 2015), previously employed for similar chrysin-loaded nanoparticle systems by Giacomeli et al. (Giacomeli et al., 2020). The analysis was carried out on an Agilent 1100 series system (Santa Clara, CA, United States), equipped with a quaternary pump (G1311A), an autosampler (G1329A), a column thermostat (G1330B), and a diode array detector (DAD—G1315B). The chromatographic runs were performed in an RP C18 column (250 mm × 4.60 mm, 5 µm), using a mobile phase composed of acetonitrile (ACN) and water containing 0.1% formic acid (70:30) at a flow rate of 1.0 mL/min and an injection volume of 30 µL. All samples and the mobile phase were filtered through a 0.45 µm filter. The detection was evaluated at a wavelength of 280 nm. The method was partially validated according to ICH Q2 (R2) guidelines (International Council for Harmonisation of Technical Requirements for Pharmaceuticals for Human Use ICH, 2023) and was linear in the concentration ranges of 0.5–12.5 μg/mL (r^2^ = 0.9963) and 10–50 μg/mL (r^2^ = 0.9981). Selectivity was confirmed by the absence of interfering peaks at the retention time of chrysin (4 min) in chromatograms of blank nanocapsules. Precision, expressed as relative standard deviation (RSD), was 0.93% for repeatability (n = 6) and 0.98% for intermediate precision (n = 12, evaluated on a different day by a different analyst), and accuracy, evaluated by recovery testing at three concentration levels, ranged from 99.97% to 100.73%.

### 2.3 Nanocapsule suspension

Suspensions of nanocapsules containing chrysin were produced using the interfacial deposition of preformed polymers technique (Fessi et al., 1989), as demonstrated in Figure 1. In brief, an organic phase was prepared by combining PCL (0.1 g), Span® 80 (0.077 g), Capryol® 90 (0.3 g), acetone (27 mL), and chrysin (5 mg), which was then kept under magnetic stirring at 40 °C for 40 min to ensure complete dissolution. Subsequently, this organic phase was injected into an aqueous dispersion containing Tween® 80 (0.077 g) in water (53 mL), and the resulting mixture was magnetically stirred for 10 min at room temperature. The solvent was then removed via rotary evaporation under reduced pressure until a final volume of 10 mL was achieved, yielding a chrysin concentration of 0.5 mg/mL (designated NC-C). For comparative purposes, blank nanocapsules without chrysin were also prepared (referred to as NC-B).

**FIGURE 1:**
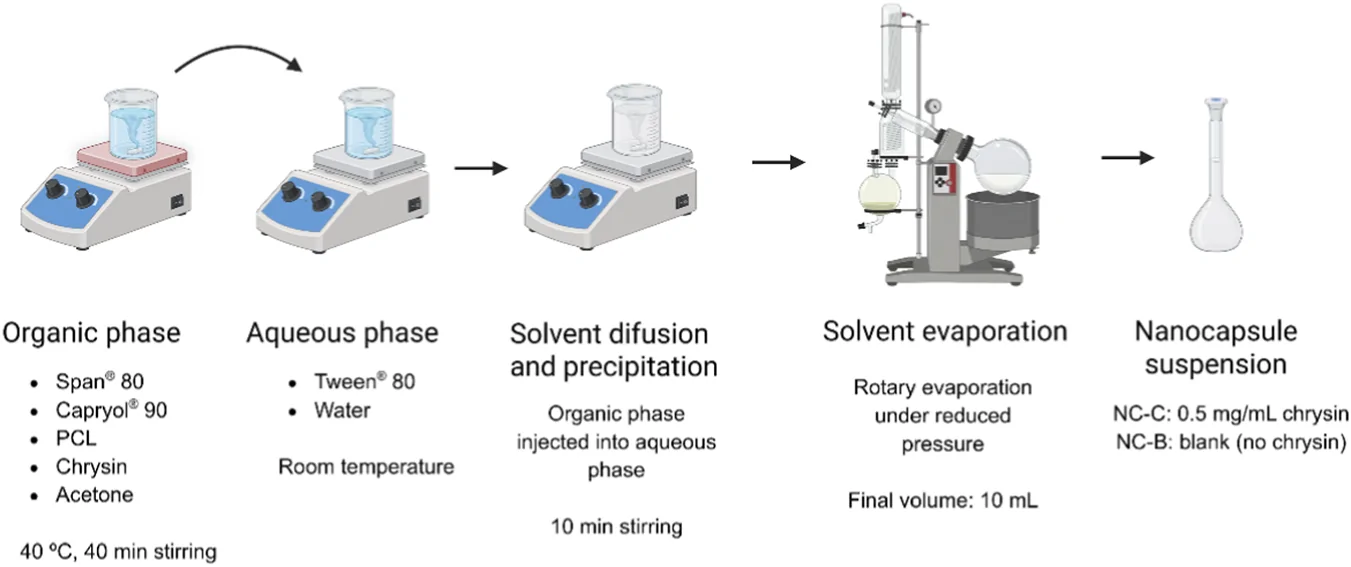
Schematic illustration of the interfacial deposition technique for preformed polymers.

### 2.4 Physicochemical characterization

#### 2.4.1 pH

The pH of the nanocapsule dispersions was measured directly in the formulations using a calibrated potentiometer (SevenEasy®, Mettler Toledo, Singapore), with all readings performed at ambient temperature (25 ± 1 °C).

#### 2.4.2 Particle size, polydispersity index (PDI), and zeta potential

The average particle diameters and polydispersity indices (PDI) were determined via photon correlation spectroscopy, following dilution of an aliquot of each dispersion in purified water at a ratio of 1:500, using a Zetasizer Nanoseries instrument (Malvern Instruments, Cambridge, United Kingdom). Zeta potential was assessed through microelectrophoresis conducted on the same equipment, after sample dilution with saline at 10 mM (1:500).

#### 2.4.3 Drug content and encapsulation efficiency (EE%)

The concentration of chrysin was measured using the HPLC method previously outlined (Section 2.2). For sample preparation, 2 mL of the formulation was diluted in 10 mL of methanol to dissolve the polymer. The resulting solution was sonicated for 30 min to ensure complete extraction of the drug, and then centrifuged at 4000 RPM for 15 min. This was then followed by a second dilution step using 2.5 mL of the supernatant and acetonitrile, after which the volume was brought to a final 10 mL, achieving a final concentration of 25 μg/mL. Subsequently, the samples were filtered through a 0.45 µm PTFE filter before injection into the HPLC system. To determine encapsulation efficiency (EE%), a 300 µL portion of the nanocapsule suspension was subjected to ultrafiltration/centrifugation using centrifugal filter devices (Amicon® 10,000 MW, Millipore, Burlington, MA, United States) at 2200×g for 10 min. The concentration of free chrysin was measured in the ultrafiltrate. The EE% was then calculated from the difference between the total and free drug concentrations, using the aforementioned HPLC method.

### 2.5 Scanning electron microscopy

Scanning Electron Microscopy was carried out to characterize the morphology of NC-C, NC-B, and free chrysin (FC), using a MIRA3 field-emission scanning electron microscope (TESCAN ORSAY HOLDING, Czech Republic). Before imaging, the nanocapsule dispersions were diluted in purified water at a 1:500 ratio, whereas free chrysin was analyzed directly as a dry powder.

### 2.6 *In vitro* drug release

The *in vitro* drug release profile of chrysin from the nanocapsules was assessed using the dialysis diffusion method. A 4 mL aliquot of NC-C (equivalent to 2.0 mg of drug) was placed into cellulose dialysis tubing with a molecular weight cut-off of 10,000 Da (SpectraPor 7, Sigma Aldrich, São Paulo, Brazil). The dialysis bag was then immersed in 400 mL of release medium, consisting of acetate buffer at pH 5.5 and 1% sodium lauryl sulfate, maintained at 37.0 ± 1 °C under constant stirring at 50 rpm. At designated time points (1h, 2h, 4h, 6h, 8h, 10h, 12h, 24h, 36h, 48h, 72h and 120 h), samples of the external medium were withdrawn and analyzed by HPLC, utilizing the method previously described in Section 2.2, to determine the percentage of cumulative drug released. The volume of release medium was replenished after each sampling to maintain sink conditions. For comparative purposes, an ethanolic chrysin solution (0.5 mg/mL) was also tested under identical conditions.

The release profiles of NC-C and FC were fitted to zero-order, first-order, Higuchi, and Korsmeyer–Peppas kinetic models using Microsoft Excel® (Version 365 Plus – 2021). Linear regression analyses were performed using the cumulative percentage of chrysin released ( $Q_{t}$ ) versus time for the zero-order model, $Q_{t}$ versus the square root of time for the Higuchi model, and the natural logarithm of $Q_{t}$ versus time for the first-order model. For the Korsmeyer–Peppas model, the released fraction ( $f_{t}=Q_{t}/100$ ) and time were converted to base-10 logarithms, and $\log\left(f_{t}\right)$ was plotted against $\log\left(t\right)$ . All experimental time points were included in the regression analyses. The release exponent ( n ) was obtained from the slope of the Korsmeyer–Peppas regression. Model fitting was evaluated using the coefficient of determination ( $r^{2}$ ), and the model with the highest $r^{2}$ was considered to provide the closest mathematical description of the experimental release profile.

The models were expressed as follows:

$$Q_{t}=k_{0}t+b$$

for the zero-order model;

$$Q_{t}=k_{H}t^{1/2}+b$$

for the Higuchi model;

$$\ln\left(Q_{t}\right)=k_{1}t+b$$

for the first-order model; and

$$\log\left(f_{t}\right)=\log\left(k_{KP}\right)+n\log\left(t\right)$$

for the Korsmeyer–Peppas model.

In these equations, $Q_{t}$ represents the cumulative percentage of chrysin released at time t , $f_{t}$ represents the released fraction, $k_{0}$ , $k_{H}$ , $k_{1}$ , and $k_{KP}$ are the respective kinetic constants, b is the regression intercept, and n is the release exponent.

### 2.7 *In vitro* antioxidant activity

The antioxidant activity of NC-C was assessed by measuring its capacity to neutralize the synthetic radical 2,2′-azinobis (3-ethylbenzothiazoline-6-sulfonic acid) (ABTS^+^), following the protocol described by Re et al. (1999) with certain adjustments. The ABTS^+^ radical solution was generated by combining a 7 mM ABTS stock solution with 140 mM sodium persulfate, left to react for 12 h prior to the assay. FC solution in methanol (0.5 mg/mL), NC-C, and NC-B were diluted in distilled water to achieve concentrations ranging from 0.625 to 50 μg/mL, and then incubated with the ABTS solution at room temperature for 30 min in the dark. An ABTS^+^ solution incubated under identical conditions served as the negative control, while an ascorbic acid solution was used as the positive control. Absorbance readings were taken at 734 nm using a spectrophotometer. Additionally, the same treatments at all tested concentrations were diluted in water instead of ABTS^+^ solution, and their absorbances were also recorded to serve as blanks, accounting for any interference caused by the opalescence of the nanocapsules during measurements. Radical scavenging activity was calculated as the percentage of inhibition relative to the negative control, according to the following equation:

$$\text{Scavenging Capacity }\left(\%\right)=100-\left[\left(\text{Abs }A\;-\text{ Abs }B\right)\;/\text{ Abs }C\right]\times 100$$

Where Abs A corresponds to the absorbance of the sample incubated with ABTS^+^, Abs B is the absorbance of the blank, and Abs C is the absorbance of the negative control.

### 2.8 *In vitro* antifungal activity

The antifungal activity of chrysin-loaded nanocapsules was evaluated using the broth microdilution protocol established by the European Committee on Antimicrobial Susceptibility Testing (EUCAST) (Guinea et al., 2023). The *C. albicans* strain obtained from the Collection of Pathogenic Fungi at the Oswaldo Cruz Foundation was cultured on Sabouraud dextrose agar (SDA; Becton Dickinson, Sparks, MD, United States) at 28 °C for 7 days. Colonies were subsequently subcultured onto fresh SDA and incubated at 37 °C for 24 h. Fungal suspensions were adjusted to a 0.5 McFarland standard in sterile saline and diluted 1:10 in RPMI-1640 medium buffered with MOPS and supplemented with 2% glucose (pH 7.0; Sigma-Aldrich, St. Louis, MO, United States).

Two-fold serial dilutions (ranging from 2.5 to 50 μg/mL) of NC-C, NC-B, and FC dissolved in DMSO were prepared in complete RPMI-1640 medium. In flat-bottom 96-well microplates, 100 µL of each test solution was mixed with 100 µL of the *C. albicans* inoculum. Growth control wells consisted of RPMI-1640 medium and the fungal inoculum. A background control, containing each test solution without fungal inoculum, was included to correct for the intrinsic optical interference of the nanocapsule suspensions. Microplates were incubated at 37 °C for 48 h.

Fungal growth inhibition was first determined spectrophotometrically at 405 nm (Multiskan™ FC, Thermo Scientific, Waltham, MA, United States) and calculated as:

$$\text{Growth }\left(\%\right)=\left[\left(\text{ODtest }-\text{ ODbackground}\right)\;/\;\left(\text{ODgrowth control }-\text{ ODbackground}\right)\right]\times 100$$

Growth inhibition (%) was calculated as 100 − Growth (%). For each formulation and each of three independent replicates, the concentrations required to achieve 70%, 80%, and 90% growth inhibition (IC_70_, IC_80_, IC_90_) and complete growth inhibition (IC_100_) were determined by linear interpolation, assuming a proportional response from the origin, using the lowest tested concentration at which each respective threshold was first attained (target concentration = threshold [%] × reference concentration/inhibition [%] at the reference concentration). Mean ± SD values across replicates are reported.

### 2.9 Spray device

The chrysin nanocapsule suspensions were put in a spray device to facilitate its topical application. To determine the chrysin content per spray, 12 actuations were discharged into a 10 mL volumetric flask. The solution was then diluted as described in Section 2.4 (Drug content and encapsulation efficiency) to achieve a final chrysin concentration of approximately 26.91 μg/mL. The final solution was analyzed by HPLC according to the previously described method.

Uniformity of delivered doses was evaluated by collecting 10 individual spray actuations and quantifying the chrysin content in each spray using the previously described HPLC method. From these results, the mean drug content, standard deviation (SD), and relative standard deviation (RSD) among the actuations were calculated.

The physicochemical stability of the nanocapsules following spraying was also evaluated by monitoring particle size and PDI after successive actuations, as described previously in Section 2.4 (Particle size, polydispersity index (PDI), and zeta potential).

The spray pattern was assessed by discharging one actuation of NC-C onto plain paper (15.4 cm in diameter) positioned 5 cm from the nozzle. The resulting deposition area was photographed and analyzed using ImageJ software (NIH, United States); the scale was calibrated using the known paper diameter, and, after appropriate thresholding, the number of discrete droplets, total deposition area, and percentage of the paper surface covered by the spray (%Area) were determined using the Analyze Particles function.

The run-off time of NC-C was assessed by discharging a single spray actuation onto a watch glass positioned vertically (90° from horizontal) and recording, with a chronometer, the time required for the formulation to completely drain off the surface. Measurements were performed in triplicate (n = 3).

The occlusive effect of NC-C was evaluated using the *in vitro* occlusion test as applied by Marchiori et al. (Marchiori et al., 2023). A sufficient volume of NC-C was applied onto a semipermeable membrane covering a container filled with distilled water, and weight loss due to water evaporation was recorded gravimetrically at 30 min, 4, 6, 8, and 24 h, alongside an uncovered control. The occlusion factor (F) was calculated based on the cumulative weight loss over 24 h as:

$$F\;\left(\%\right)=\left[\left(A\;-\;B\right)\;/\;A\right]\times 100$$

where A is the total water loss (g) of the uncovered control over 24 h, and B is the mean total water loss (g) of the membranes covered with NC-C (n = 3) over the same period.

### 2.10 Statistical analysis

All formulations were independently prepared and evaluated in triplicate (n = 3). Data are expressed as mean ± SD or standard error of the mean (SEM). Statistical differences were evaluated using one/two-way analysis of variance (ANOVA), followed by Šídák’s or Tukey’s multiple comparisons test, depending on the study design. Differences were considered statistically significant at *p* < 0.05. Statistical analyses and graphs were performed using GraphPad Prism® (version 8).

## 3 Results

### 3.1 Physicochemical characterization

The NC-C dispersion presented an eggshell-white coloration with a subtle blue opalescence, characteristic of the Tyndall effect. The absence of visible precipitation or any other signs of physical instability suggests that the formulation possesses adequate colloidal stability. The loaded nanocapsules exhibited a pH of 5.679 ± 0.15, a mean particle size of 207.6 ± 4.63 nm, a PDI of 0.174 ± 0.04, and a zeta potential of −10.44 ± 0.94 mV. NC-B demonstrated similar physicochemical characteristics, with a pH of 5.055 ± 0.19, mean particle size of 208.94 ± 9.95 nm, PDI of 0.176 ± 0.045, and zeta potential of −12.6 ± 2.12 mV. No statistically significant differences were observed between blank and drug-loaded nanocapsules (*p* > 0.05), suggesting that the incorporation of chrysin did not substantially alter the physicochemical properties of the nanocapsule system. Table 1 summarizes the physicochemical characterization of the nanocapsules.

**TABLE 1:** Physicochemical characterization of chrysin-loaded nanocapsules (NC-C) and blank nanocapsules (NC-B), including particle size, PDI, zeta potential, pH, drug content and encapsulation efficiency.

Formulation	Particle size (nm)	PDI	Zeta potential (mV)	pH	Drug content (%)	EE%
NC-C	207.6 ± 4.63	0.174 ± 0.04	−10.44 ± 0.94	5.679 ± 0.15	99.36 ± 7.73	87.28 ± 0.77%
NC-B	208.94 ± 9.95	0.176 ± 0.045	−12.6 ± 2.12	5.055 ± 0.19	-	-

The drug content was determined to be 99.36 ± 7.73%, which is close to the theoretical value of 0.5 mg/mL, indicating excellent recovery of the drug during the formulation process and minimal losses during preparation. The EE% was 87.28 ± 0.77%, reflecting the high capacity of the polymeric matrix to retain chrysin within the nanocapsule core.

### 3.2 Scanning electron microscopy

Scanning electron microscopy images (Figure 2) revealed predominantly rounded to irregular structures with a relatively smooth surface and a heterogeneous size distribution for both NC-C and NC-B. Some structures appeared individually dispersed, whereas pronounced aggregation was observed in areas with a higher particle density. The observed morphology was consistent with the particulate structure of the formulation. Free chrysin in its raw powder form showed highly irregular, angular, and blocky particles with a rough, crystalline surface texture.

**FIGURE 2:**
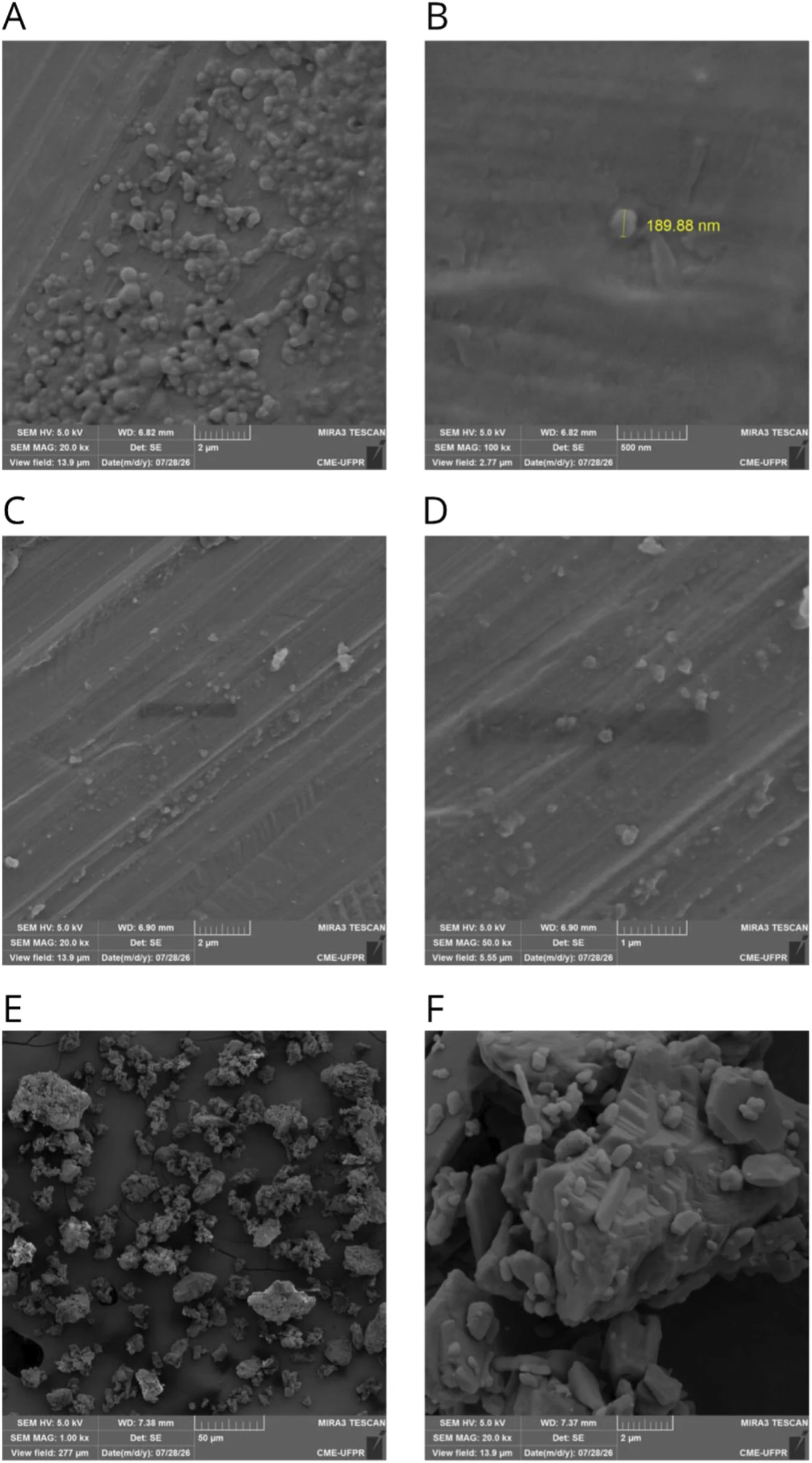
Scanning electron microscopy images comparing chrysin-loaded nanocapsules, blank nanocapsules and free chrysin. **(A,B)** NC-C shown at 2 μm and 500 nm scale bars, respectively. **(C,D)** NC-B shown at 2 μm and 1 µm scale bars, respectively. **(E,F)** Free chrysin powder shown at 50 μm and 2 µm scale bars, respectively.

### 3.3 *In vitro* drug release

The *in vitro* drug release test showed that, overall, the chrysin-loaded nanocapsules and free chrysin demonstrate contrasting release behaviors. NC-C displays a sustained-release behavior characterized by a gradual controlled increase in drug concentration over time. The release starts at a low percentage within the first hour (∼4%) and steadily rises over the initial 10 h (∼18%). Between 24 and 48 h, the release rate accelerates steadily from roughly 22–38%, eventually reaching a maximum cumulative release of approximately 68% at 120 h.

In contrast, FC shows a pronounced initial burst effect, releasing roughly 32% of the active compound within the first hour and maintaining near 40% release between 4 and 6 h. This initial release from FC was significantly higher than from NC-C (*p* < 0.05) at 1, 2, 4, 6, and 12 h, reflecting the immediate availability of the non-encapsulated drug. After this initial phase, the release profile plateaus and fluctuates between 30% and 40% up to 48 h, followed by a late peak near 55% at 120 h. From 24 h onward, no significant differences were observed between NC-C and FC (*p* > 0.05), as the sustained release from NC-C progressively approached and ultimately slightly exceeded that of FC, reaching a maximum cumulative release of approximately 68% at 120 h (Figure 3).

**FIGURE 3:**
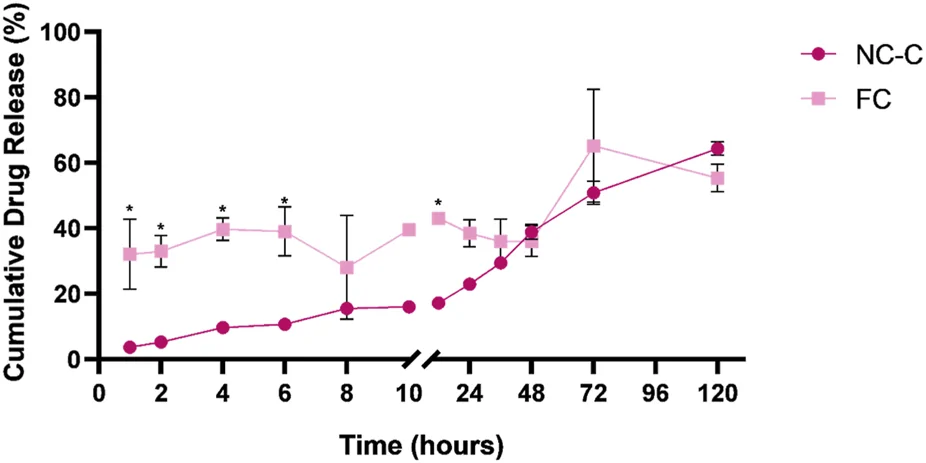
Cumulative release percentage (%) of chrysin-loaded nanocapsules (NC-C) compared to free chrysin ethanolic solution (FC) over a 120-h period. NC-C demonstrates a sustained release profile, whereas FC exhibits an initial burst release. The x-axis is interrupted between 10 and 24 h to accommodate the more frequent early sampling points. Results represent the mean ± SD of three independent experiments. Statistical analysis was performed using two-way ANOVA followed by Šídák’s multiple comparisons test. Asterisks (*) represent significant statistical difference (*p* < 0.05) between NC-C and FC at the corresponding time point.

To investigate the mechanism underlying drug release, the cumulative release data were fitted to zero-order, first-order, Higuchi, and Korsmeyer–Peppas kinetic models. The NC-C release profile was best described by the Higuchi model ( $r^{2}=0.959$ ), closely followed by the zero-order model ( $r^{2}=0.955$ ). The Korsmeyer–Peppas model showed a comparatively lower fit ( $r^{2}=0.895$ ) and yielded a release exponent of n=0.468 . Considering the approximately spherical geometry of the nanocapsules, this value lies within the range associated with anomalous transport ( 0.43<n<0.85 ), although it remains close to the theoretical limit for Fickian diffusion. Together with the higher fit obtained for the Higuchi model, this finding suggests that chrysin release was predominantly diffusion-driven, with a possible secondary contribution from polymer relaxation and drug partitioning across the oily core–polymer–aqueous interfaces. The comparatively poor fit of the first-order model ( $r^{2}=0.715$ ) further indicates that the release behavior was not adequately described by this model. None of the four models adequately described the FC profile, with $r^{2}$ values ranging from 0.582 for Korsmeyer–Peppas to 0.647 for zero-order kinetics.

### 3.4 *In vitro* antioxidant activity

At lower concentrations (0.625–2.5 μg/mL), NC-C exhibited significantly higher scavenging activity than both NC-B and FC, with the most pronounced differences observed at 2.5 μg/mL (*p* < 0.0001 vs. NC-B; *p* = 0.0037 vs. FC). From 5 μg/mL, NC-C and FC showed comparable antioxidant activities (*p* > 0.05 for all concentrations), while NC-B consistently displayed significantly lower scavenging capacity across all tested concentrations (Figure 4).

**FIGURE 4:**
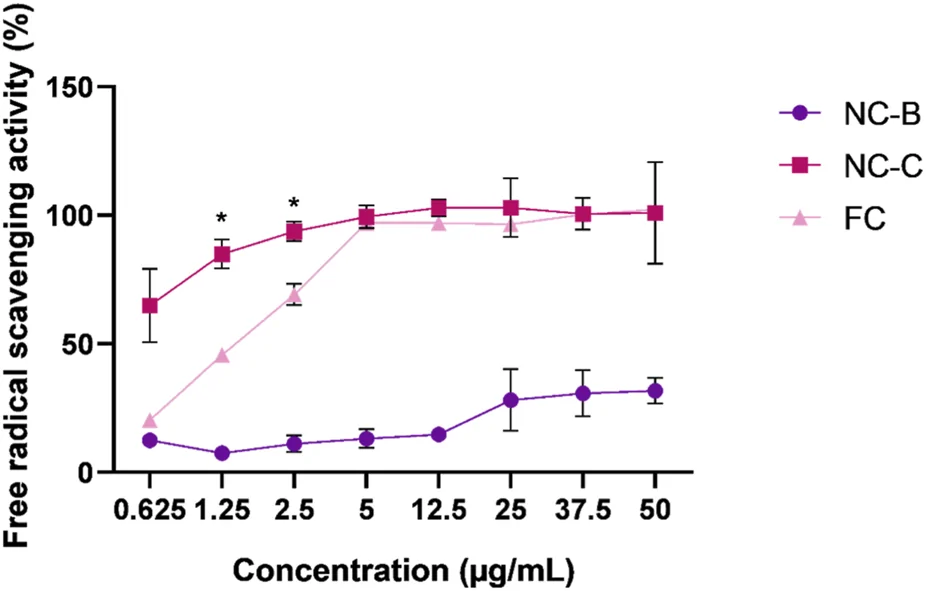
Scavenging activity of FC, NC-C, and NC-B tested at concentrations ranging from 0.625 μg/mL to 50 μg/mL. Results represent the mean ± SD (n = 3). Asterisks (*) denote statistically significant differences (*p* < 0.05) between NC-C and FC by two-way ANOVA followed by Tukey’s *post hoc* test.

### 3.5 *In vitro* antifungal activity

The *in vitro* antifungal activity against *C. albicans* was evaluated for NC-C, FC, and NC-B across a concentration range of 2.5–50 μg/mL. To characterize antifungal potency, the concentrations required to achieve 70%, 80%, and 90% growth inhibition (IC_70_, IC_80_, IC_90_), and complete growth inhibition (IC_100_), were determined for each formulation (Figure 5; Table 2). NC-C and NC-B required substantially lower concentrations than FC to reach equivalent inhibition levels at every endpoint evaluated: NC-C was approximately 7.7-, 7.7-, 5.0-, and 5.5-fold more potent than FC at the IC_70_, IC_80_, IC_90_, and IC_100_ endpoints, respectively, while NC-B was approximately 11.5-, 11.5-, 7.1-, and 4.9-fold more potent at the same endpoints.

**FIGURE 5:**
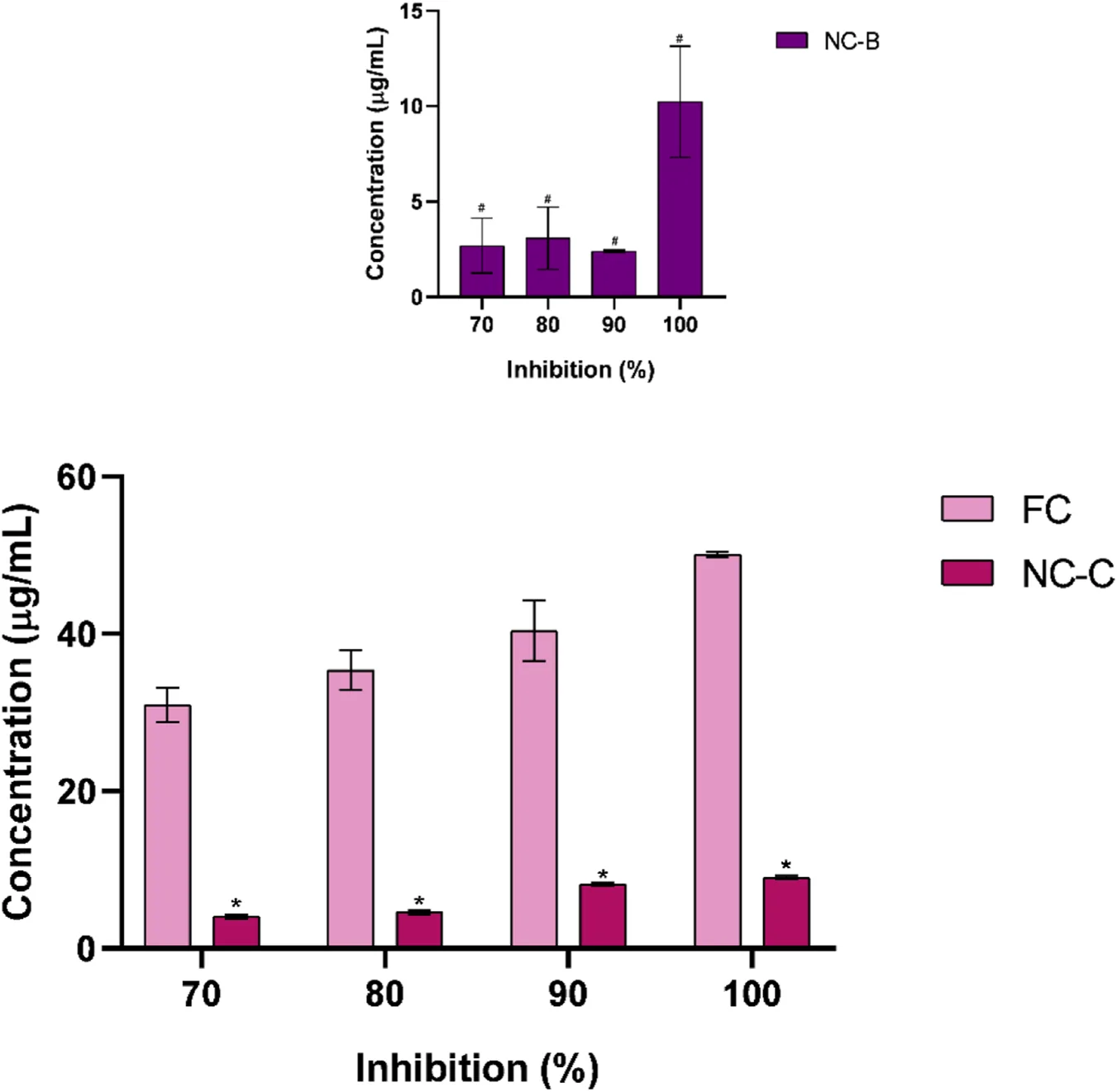
Inhibitory (IC_70_, IC_80_, IC_90_), and IC_100_ concentrations of NC-B (top) and NC-C (bottom) compared with free chrysin (FC) against *C. albicans*. Data are presented as mean ± standard deviation (n = 3). Statistical significance compared to FC is indicated by (#) for NC-B and (*) for NC-C, determined by two-way ANOVA followed by Šídák’s multiple comparisons test (*p* < 0.05).

**TABLE 2:** Inhibitory concentrations (IC_70_, IC_80_, IC_90_) and concentration for complete growth inhibition (IC_100_) of NC-C, NC-B, and FC against *C. albicans*, interpolated from individual replicate dose-response data (mean ± SD, n = 3).

Formulation	IC_70_ (µg/mL)	IC_80_ (µg/mL)	IC_90_ (µg/mL)	IC_100_ (µg/mL)
NC-C	4.03 ± 0.25	4.61 ± 0.29	8.14 ± 0.19	9.04 ± 0.21
NC-B	2.70 ± 1.43	3.08 ± 1.63	5.68 ± 0.04	10.24 ± 2.91
FC	30.97 ± 2.20	35.39 ± 2.52	40.41 ± 3.85	50.12 ± 0.36

### 3.6 Spray device

The mean drug content recovered per actuation was 106.75 ± 6.17 µg, corresponding to 118.5% of the theoretical dose. The relative standard deviation was 5.78%, indicating low inter-actuation variability. These results demonstrate that the topical spray device provides consistent and reproducible drug delivery per actuation, which is essential for ensuring reliable dosing in clinical use.

Despite the stress associated with spray processing, the particle size and PDI of both NC-B and NC-C nanocapsules remained largely unchanged, with no significant statistical difference (*p* > 0.05). Before processing, NC-B exhibited an initial mean particle size of 229.56 ± 28.34 nm and a PDI of 0.172 ± 0.02, which shifted only minimally to 200.06 ± 9.94 nm and 0.106 ± 0.01 following spraying. Similarly, NC-C demonstrated excellent physical stability throughout the process, maintaining its narrow size distribution with pre- and post-spray mean sizes of 230.18 ± 11.10 nm and 225.09 ± 36.67 nm, respectively, alongside corresponding PDI values of 0.231 ± 0.04 and 0.184 ± 0.07. The preservation of these physicochemical attributes confirms that spray processing did not induce significant particle aggregation or disruption of the nanocarrier matrices. The summarized data can be observed in Table 3.

**TABLE 3:** Comparison of particle size and PDI for NC-B and NC-C nanocapsules pre- and post-spray processing.

Formulation	Particle size (nm)		PDI	
	Pre-spray	Post-spray	Pre-spray	Post-spray
NC-C	230.18 ± 11.10	225.09 ± 36.67	0.231 ± 0.04	0.184 ± 0.07
NC-B	229.56 ± 28.34	200.06 ± 9.94	0.172 ± 0.02	0.106 ± 0.01

At a spray distance of 5 cm, the formulation produced a ring-shaped (annular) deposition pattern, with the majority of the spray content concentrated in the outer perimeter and minimal deposition in the central region, accompanied by scattered satellite droplets beyond the main pattern, together comprising 59.5 ± 0.71 discrete particles with a total deposition area of 25.29 ± 1.43 cm^2^ (mean particle size 0.425 ± 0.018 cm^2^), corresponding to 13.54 ± 1.10% coverage of the 15.4 cm-diameter paper target. This annular distribution is consistent with a hollow-cone spray pattern, commonly observed in pump-actuated devices, in which droplets are preferentially atomized toward the periphery of the spray cone rather than along its central axis. Figure 6 shows the spray pattern.

**FIGURE 6:**
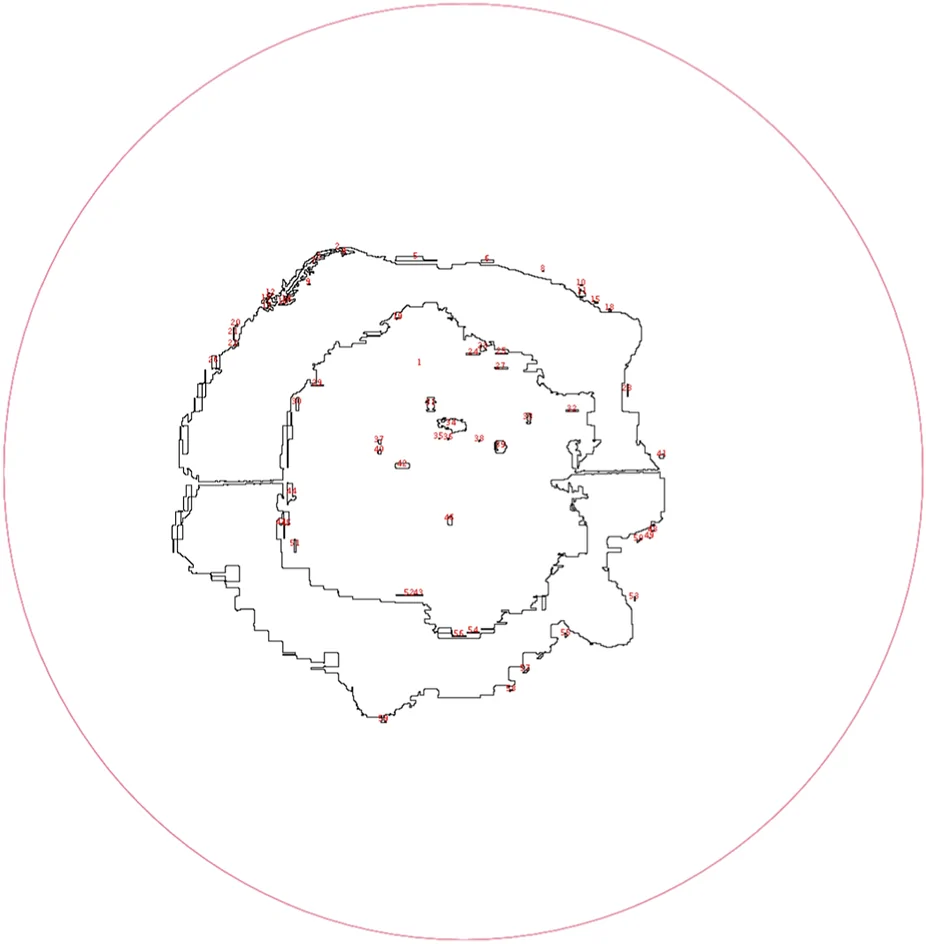
Spray pattern of NC-C on the paper substrate. The outer circle delineates the edge of the paper target; the outlined shapes correspond to the individual droplets identified by particle analysis, showing a central ring-shaped deposition surrounded by scattered satellite droplets.

The formulation presented a run-off time of 1.3 ± 0.1 s, indicating that NC-C drains rapidly from an inclined surface without prolonged adherence, which is consistent with a low-viscosity, easily spreadable topical spray. The occlusion factor of NC-C over 24 h was 3.21 ± 3.92%, indicating a minimal occlusive effect, consistent with the absence of a film-forming polymer in the formulation.

## 4 Discussion

The physicochemical properties of a nanocapsule formulation are fundamental determinants of its performance, stability, and safety for topical application. In the present study, the mean particle size was within the nanometric range, a critical attribute for dermal delivery. Nanoparticles of this size are known to enhance skin permeation by facilitating passage through the stratum corneum and enabling targeted delivery to deeper epidermal layers, while simultaneously minimizing systemic absorption and associated off-target effects (Roy et al., 2020). The low PDI values reflect a homogeneous population with a narrow size distribution, indicating high reproducibility of the preparation method and ensuring consistent application properties, a prerequisite for predictable therapeutic outcomes.

The negative zeta potential values suggest moderate physical stability, as the surface charge is expected to generate electrostatic repulsion between particles, thereby reducing the likelihood of aggregation over time. This colloidal stability is essential for preserving product uniformity during storage and use, directly impacting shelf-life and patient compliance. Furthermore, the pH of the formulation was compatible with the skin’s physiological pH range (approximately 4.5–5.5), which is crucial not only for preventing local irritation and preserving the skin barrier function but also for maintaining the chemical integrity of both the encapsulated drug and the polymeric matrix (Mijaljica et al., 2025).

A notably high encapsulation efficiency (EE%) was achieved, which holds particular significance for topical formulations. High EE% ensures that a substantial proportion of the drug remains entrapped within the nanocapsules, thereby protecting it from chemical and enzymatic degradation, enabling controlled release at the application site, and improving cutaneous bioavailability. Additionally, the polymeric coating may promote prolonged retention on the skin surface, favoring sustained drug release and reducing the required frequency of application, which enhances patient adherence (El-Hussien et al., 2021; Guterres et al., 2007). The low standard deviation associated with the EE% further attests to the consistency and reliability of the encapsulation process, reinforcing batch-to-batch reproducibility—a cornerstone of pharmaceutical development and regulatory compliance.

Chrysin-loaded PCL nanocapsules markedly delayed drug transfer to the release medium compared with free chrysin: cumulative release reached only 22% for NC-C versus 38% for FC after 24 h, and approximately 68% for NC-C versus 55% for FC by 120 h. This profile indicates substantial initial retention followed by a gradual, sustained release phase. The pronounced retardation is likely attributable to several factors: the lipophilic nature of chrysin favors its partitioning into the Capryol® 90 oily core, while the PCL shell and surfactant-rich interface provide additional diffusional barriers to drug transfer into the aqueous medium.

Consistent with a predominantly diffusion-driven process, the release profile of NC-C was best described by the Higuchi model ( $r^{2}=0.959$ ). The Korsmeyer–Peppas model yielded a release exponent of n=0.468 , which, considering the approximately spherical geometry of the nanocapsules, is slightly above the theoretical value associated with Fickian diffusion ( n=0.43 ). This result suggests anomalous transport with a dominant diffusional contribution and a possible secondary influence of drug partitioning across the oily core–polymer–aqueous interfaces or polymer relaxation. However, because the Korsmeyer–Peppas model was fitted to the complete release profile, the n value should be regarded as supportive rather than conclusive evidence of the release mechanism. In contrast, none of the evaluated kinetic models adequately described the FC profile ( $r^{2}=0.582–0.647$ ). This poor fit is consistent with its fluctuating release behavior, which may reflect the combined influence of chrysin dissolution, precipitation, adsorption to the dialysis membrane, and membrane diffusion rather than release from a structured carrier.

This sustained behavior is consistent with previously reported polymeric delivery systems for chrysin. Firouzi-Amandi et al. observed that chrysin-loaded PLGA-PEG nanoparticles exhibited slow, controlled release at pH 7.4, reaching approximately 65% cumulative release over 120 h following an initial burst within the first 8 h (Firouzi-Amandi et al., 2018). Similarly, Ezzat et al. reported a cumulative release of 56.59% ± 0.81% from chrysin-loaded PLGA nanoparticles after 72 h at pH 7.4, significantly exceeding the 31.84% ± 2.12% released from a pure chrysin suspension, a difference attributed to drug amorphization and non-Fickian transport involving diffusion and polymer relaxation (Ezzat et al., 2025). In another polymeric approach, Ragab et al. demonstrated progressive, controlled release from chrysin-chitosan nanoparticles, with cumulative values of 28.86% ± 0.057% at 4 h, 57.88% ± 0.0057% at 6 h, 68.43% ± 0.0152% at 10 h, and 85.2% ± 1% at 24 h (Ragab et al., 2022). More recently, Kotb et al. showed that chrysin-loaded PLGA nanoparticles sustained release over 50 h (Kotb et al., 2025).

The gradual increase observed in NC-C after 24 h may reflect the presence of distinct drug populations: a readily available fraction near the particle surface and a more strongly retained fraction sequestered within the oily core. While polymer relaxation may contribute to the later release phase, extensive PCL degradation within 120 h is unlikely without direct supporting evidence.

The fluctuating release profile observed for FC between 8 and 72 h should be interpreted with caution. Since free chrysin was not encapsulated, its rapid initial diffusion does not represent a true burst release, but rather the transfer of an initially soluble fraction, which may be followed by non-specific interaction of chrysin with the dialysis membrane, a limitation previously described for dialysis-based release methods, particularly for compounds with high membrane affinity (Yu et al., 2019), producing transient, reversible reductions in the apparent dissolved drug concentration. Additionally, given the extended duration of the assay (120 h), partial degradation or instability of chrysin in solution cannot be ruled out as a contributing factor to the irregular release pattern observed for the unformulated compound.

The finding that nanoencapsulation significantly enhances the antioxidant efficacy of chrysin, particularly at lower concentrations (0.625–2.5 μg/mL), is strongly supported by previous reports on the nanoscale modification of this flavonoid (Khalid and Naseem, 2022; Sulaiman et al., 2018). Using the ABTS assay to evaluate total antioxidant capacity (TAC), one study conducted by Khalid et al. demonstrated that chrysin nanoparticles exhibited significantly greater radical-scavenging activity than free chrysin at lower concentrations (5–25 mM), while the differences between the two formulations became less pronounced at higher concentrations (Khalid and Naseem, 2022). This trend is further corroborated by Sulaiman et al., who assessed free radical scavenging via the DPPH method across a concentration range of 40–200 μg/mL and reported markedly enhanced antioxidant activity for nanoencapsulated chrysin compared with its free counterpart (Sulaiman et al., 2018).

Nanoencapsulation enhanced the antifungal efficacy of chrysin against *C. albicans* compared with the unformulated compound, with NC-C achieving substantially lower inhibitory and fungicidal concentrations, suggesting improved aqueous solubility and bioavailability. However, this response cannot be attributed exclusively to chrysin, as NC-B also inhibited *C. albicans* throughout the concentration range, indicating that the nanocapsule components possess intrinsic antifungal activity. The superior activity of NC-C should therefore be interpreted as a combined effect of chrysin and the nanocarrier rather than a direct consequence of improved solubility alone. The blank-carrier effect likely originates from the surfactant-rich lipophilic interface (Tween® 80, Span® 80, Capryol® 90), which may interact with the fungal cell envelope (Aonofriesei, 2024; Duarte et al., 2023). However, attributing this effect to specific excipients remains speculative, as *Candida* species can hydrolyze Tween® 80 through extracellular lipolytic activity (Slifkin, 2000). Testing the isolated excipients at relevant concentrations would be necessary to identify the active component.

The activity of free chrysin is consistent with previous reports identifying flavonoids as natural antifungal agents. Wang et al. (Wang and Li, 2026) demonstrated that chrysin inhibits *C. albicans* biofilm formation and hyphal development, though the present planktonic assay was not designed to assess these specific effects. Further investigation using dedicated hyphal-transition and antibiofilm assays would be valuable to determine whether nanoencapsulation preserves or potentiates these mechanisms. Plausible hypotheses for chrysin’s activity include membrane perturbation, interference with ergosterol homeostasis, and inhibition of adhesion-related pathways, all of which warrant direct experimental validation.

The IC_70_, IC_80_, IC_90_, and IC_100_ values (Table 2) provide direct, quantitative confirmation of the potency enhancement afforded by nanoencapsulation: NC-C was approximately 5- to 8-fold more potent than free chrysin across the inhibition endpoints evaluated, consistent with the improved solubility and bioavailability conferred by the nanocapsule. Notably, NC-B showed a comparable or even greater potency advantage over FC at most endpoints, reinforcing the interpretation that the nanocapsule excipients themselves contribute meaningfully to the antifungal effect, independent of the encapsulated chrysin. Direct clinical benchmarking of NC-C was not possible in the absence of a standard antifungal control. Nonetheless, the IC_70_–IC_100_ values obtained (4.03–9.04 μg/mL) can be tentatively contextualized against previously reported values. Wild-type *C. albicans* strains are typically susceptible to fluconazole within the 0.5–2 μg/mL range according to EUCAST epidemiological cutoff values (Guinea et al., 2023), placing NC-C above this range, though considerably below the sessile MIC_50_ (128 μg/mL) reported for free chrysin against *C. albicans* biofilms (Wang and Li, 2026) and within the same order of magnitude as minimum inhibitory concnentration values reported for other nanoparticle-encapsulated natural antifungal compounds, such as lawsone-loaded mesoporous silica nanoparticles (0.31 to >5 μg/mL) (Nikoomanesh et al., 2025). These comparisons should be interpreted with caution given differences in assay format, strain panel, and endpoint criteria across studies, reinforcing the need for head-to-head testing against a standard antifungal control in future work. Future studies incorporating conventional antifungal controls, additional reference and clinical isolates, and confirmatory minimum fungicidal concentration assays would further strengthen the biological interpretation.

The preliminary repeatability of the chrysin-loaded PCL nanocapsule (NC-C) spray formulation, evidenced by an 118.5% recovery of the theoretical dose and a relative standard deviation (RSD) of 5.78%, is highly comparable to the delivered-dose uniformity and weight consistency reported in the literature for topical metered-dose sprays. Paradkar et al. noted that an insignificant variation in the weight delivered per actuation confirms the effectiveness and reproducibility of the pump mechanism in delivering accurate doses (Paradkar et al., 2015). Maintaining reproducible actuation is especially critical for nanocarrier suspensions to prevent dosing fluctuations caused by nanoparticle sedimentation, retention in the pump chamber, or phase separation during actuation (Uronnachi et al., 2024). In metered-dose spray formulations of colloidal itraconazole, Uronnachi et al. emphasized that accurate dosing and formulation dispersion stability are paramount, as conventional topical formats suffer from highly variable dosing and poor penetration into target crevices (Uronnachi et al., 2024; Geh et al., 2019). The acceptable repeatability of NC-C indicates that the nanocapsules remained successfully dispersed and compatible with the spray device during application, preventing the nozzle clogging or inconsistent delivery often associated with poorly dispersed or highly viscous colloidal systems.

Furthermore, the mechanical stability of the NC-C colloidal system under the high shear stress of atomization is supported by the absence of significant changes in particle size and PDI following actuation. Passage through a spray nozzle subjects nanocarriers to intense shear forces, pressure gradients, and contact with device surfaces, which can compromise colloidal integrity and induce aggregation. The preservation of these critical quality attributes in NC-C aligns with the physical stability shown in optimized nanoparticle sprays, such as the voriconazole polymeric nanoparticles developed by Mumtaz et al., where nanoparticle structural integrity and spherical shape were maintained after being incorporated into the spray presentation (Mumtaz et al., 2022).

Importantly, NC-C represents a distinct formulation strategy from most previously reported antifungal and transdermal nanoparticle sprays. While Mumtaz et al. and Paradkar et al. incorporated nanocarriers or active ingredients into polymeric film-forming matrices (typically using Eudragit E100), the present NC-C formulation delivers the PCL nanocapsules as a simple aqueous suspension without an additional film-forming polymer. Polymeric film-forming sprays are designed to create a concentrated, thin layer on the skin surface that acts as a localized reservoir to prolong drug release, but they present distinct technical challenges, such as the risk of film cracking or flaking at higher polymer concentrations and the risk of localized skin irritation or bacterial growth under highly occlusive films (Paradkar et al., 2015; Mumtaz et al., 2022). In contrast, the non-film-forming suspension of NC-C avoids these occlusive film limitations, as confirmed by its negligible *in vitro* occlusion factor (3.21%), and provides a contact-free, convenient application, which is highly advantageous for application over sensitive, irregular, or friction-prone skin areas. However, as film-forming platforms are specifically utilized to establish a local reservoir that extends drug release (e.g., voriconazole release up to 24 h or clotrimazole release up to 12 h) (Paradkar et al., 2015; Mumtaz et al., 2022), further studies are required to determine if the non-film-forming NC-C presentation can maintain a sufficient residence time on the skin surface to support prolonged therapeutic efficacy.

## 5 Limitations and future perspectives

This study has some limitations that should be acknowledged. First, antifungal activity was evaluated against a single *C. albicans* reference strain from a fungal culture collection; clinical isolates, including azole- or echinocandin-resistant strains, were not tested. Given that antifungal susceptibility can vary considerably between reference and clinical strains, future studies should include a broader panel of *C. albicans* isolates, particularly clinically relevant and drug-resistant strains, to better establish the translational potential of this formulation.

Second, IC70–IC100 values were estimated by interpolation for all three formulations; direct experimental confirmation at concentrations below those tested, together with confirmatory MFC assays using standard colony-count methods, would strengthen confidence in these potency estimates.

Third, the broth microdilution assay did not include a conventional antifungal agent (e.g., fluconazole, miconazole, or clotrimazole) as a reference control. The inclusion of such a control is standard practice in antifungal susceptibility studies, including those evaluating PCL-based nanocapsules for cutaneous candidiasis (Nasr et al., 2026) and other nanoparticle-based delivery systems for natural antifungal compounds (Nikoomanesh et al., 2025; Far et al., 2026), and is essential both to validate assay sensitivity and to enable direct clinical benchmarking. While our internal comparison between NC-C, NC-B, and FC remains valid and informative, this omission limits the ability to position the present formulation relative to established antifungal therapy. Future studies should incorporate a standard antifungal control under identical assay conditions.

Finally, the proposed spray formulation was characterized in terms of its physicochemical stability and delivery uniformity, but its skin residence time and *in vivo* efficacy were not directly assessed. As the formulation does not include a film-forming polymer, future studies should evaluate its retention on the skin surface and its therapeutic performance in relevant *in vivo* models of cutaneous candidiasis, which will be essential to translate the promising *in vitro* findings reported here into clinical application.

## 6 Conclusion

In conclusion, chrysin-loaded PCL nanocapsules were successfully developed as a spray formulation for the topical treatment of cutaneous candidiasis. The nanocapsules exhibited suitable physicochemical properties, including nanometric particle size, low polydispersity, adequate surface charge, and pH compatible with skin physiology. High encapsulation efficiency and drug content were achieved, ensuring effective drug loading and recovery.

The sustained release profile demonstrated by NC-C effectively overcame the solubility limitations of free chrysin, eliminating the burst effect and providing a controlled and reproducible delivery over 120 h. In addition to this, nanoencapsulation significantly enhanced the antifungal activity against *C. albicans*, achieving substantially lower inhibitory and fungicidal concentrations than the unformulated compound, while also improving antioxidant efficacy at lower doses.

The spray device proved capable of delivering consistent and reproducible doses, supporting its potential for practical clinical use. Collectively, these results indicate that chrysin-loaded PCL nanocapsules represent a promising nanotechnology-based strategy for the management of skin candidiasis. However, further studies are still needed to fully elucidate the mechanisms underlying the enhanced antifungal activity, evaluate the formulation’s stability over extended storage periods, and assess its safety and efficacy in *in vivo* models, including skin irritation and sensitization tests, as well as clinical trials to confirm its therapeutic potential in humans. Additionally, investigations comparing this formulation with conventional antifungal treatments would be valuable to establish its place in clinical practice.

Despite these promising findings, the study remains at an early preclinical stage. The antifungal mechanism and the individual contributions of chrysin and the excipients should be better investigated. Finally, *ex vivo* skin permeation and retention, residence after spray drying, adhesion to the application site, long-term stability, and safety must be investigated. These studies will determine whether the prolonged *in vitro* release translates into sustained local exposure and therapeutic benefit. Overall, the developed chrysin-loaded PCL nanocapsule spray represents a promising topical platform, but further mechanistic, cutaneous-performance, and safety studies are required before its translational potential can be established.

## Data Availability

The raw data supporting the conclusions of this article will be made available by the authors, without undue reservation.
